# Functional outcome of stroke inpatients according to human immunodeficiency virus status: A feasibility study

**DOI:** 10.4102/ajod.v9i0.618

**Published:** 2020-03-30

**Authors:** Tasneem Hartley, Marlette Burger, Tonya M. Esterhuizen, Gakeemah Inglis-Jassiem

**Affiliations:** 1 Division of Physiotherapy, Faculty of Medicine and Health Sciences, Stellenbosch University, Cape Town, South Africa; 2 Division of Epidemiology and Biostatistics, Department of Global Health, Faculty of Medicine and Health Sciences, Stellenbosch University, Cape Town, South Africa

**Keywords:** stroke, HIV, function, activities of daily living, mobility

## Abstract

**Background:**

Stroke in human immunodeficiency virus positive (HIV+) individuals is becoming an increasing concern. Being significantly younger than typical stroke patients, the impact of functional challenges on quality of life and burden on society becomes more eminent.

**Objectives:**

This feasibility study aims to determine the requirements for a large descriptive cohort, to adequately describe the functional outcome of stroke patients with varying HIV status.

**Method:**

All stroke patients meeting the inclusion criteria were recruited over a 6-month period at a South African inpatient rehabilitation centre. Data were collected on admission and discharge using outcome measures including the Barthel Index (BI), Berg Balance Scale (BBS) and the use of assistive devices used to describe independence with activities of daily living (ADL), mobility and safety post-stroke. Statistical analysis was performed using Stata version 14.2.

**Results:**

The feasibility study identified appropriate procedures and barriers to a successful study in addition to describing preliminary data on participant demographics, relevant medical history and functional outcomes post-stroke. Limitations that affected feasibility included minimal recruitment sites, length of data collection period, timely communication of participant discharge plans and dates, and confirmation of participant HIV status. An appropriate comparison between sub-groups could not be made because of disproportionate group sizes, median age differences and no assessor blinding.

**Conclusion:**

To increase generalisability and the understanding of the unique HIV+ stroke profile, multiple recruitment sites, longer data collection periods, assessor blinding and age-matched groups with HIV status confirmation are recommended.

## Introduction

Functional outcomes may differ between stroke patients who are human immunodeficiency virus-negative (HIV−) and those who are HIV-positive (HIV+) because of differences in demographic characteristics, risk factors and disease manifestations (Heikinheimo et al. 2012; Tipping et al. 2007; Verma et al. 2012). Low- to middle-income countries, particularly in sub-Saharan Africa, have seen a rise in stroke prevalence (Benjamin et al. 2012; Zimba et al. 2017). Other than the rise in non-communicable diseases, such as hypertension and diabetes, which have been linked to an increased risk of stroke, HIV-related stroke in this region is becoming a concern (Chin 2012; Modi, Modi & Mochan 2008). Sub-Saharan Africa is said to have 52% of the global HIV+ population (UNAIDS 2016). This disproportionate amount weighs heavily on its healthcare system (Mochan, Modi & Modi 2003; Zimba et al. 2017). Furthermore, it is concerning that those with HIV-related strokes are found to be significantly younger than the typical stroke population (Heikinheimo et al. 2012; Mlay & Bakari 2012). This may pose a greater burden in sub-Saharan Africa as 34% of HIV+ people are aged between 15 and 24 years, whereas globally only 22% of the HIV population are in this age range (UNAIDS 2016). A previous study predicted that cardiovascular diseases, including stroke, are set to surpass infectious diseases as the major cause of morbidity and mortality in sub-Saharan Africa by the year 2020 (Yusuf et al. 2004). However, little is known about the mortality and, more specifically, the morbidity of HIV+ stroke patients in this region. In addition, information on the functional outcomes of HIV+ people with stroke who reside in sub-Saharan Africa and how much they differ from their typical stroke counterparts is still sparse.

Studies show that 40% – 66% of people with stroke still require assistance with activities of daily living (ADL) and mobility (Connor et al. 2004; Verma et al. 2012). People with stroke may have varying degrees of severity and symptoms of stroke, depending on the area of the brain affected. Some of the common symptoms include hemiparesis, hemisensory loss, hemineglect, dysphasia, dysarthria, ataxia, visual impairments, hearing impairments and vertigo (Markus 2012). These impairments affect function, ADL and ultimately the quality of life (Markus 2012). The inability to perform ADL impairs work ability, including the ability to remain a functional member of society, placing further strain on the sub-Saharan economy (Mochan et al. 2003). Human immunodeficiency virus itself can negatively affect a person’s physical and cognitive well-being (Dudgeon et al. 2006; Moore et al. 2011; Woods et al. 2009). The added neurological impairments caused by conditions such as stroke may make the afflicted more dependent and thereby less productive members of society (Mochan et al. 2003). More importantly, stroke or neurological conditions, in addition to the consequences of HIV, can further be detrimental to the quality of life of the affected individuals (Hughes et al. 2004; Rouillard et al. 2012).

It is therefore postulated that HIV+ stroke patients may differ from typical stroke patients. Rehabilitation post-stroke is aimed at improving the quality of life of those affected by enhancing their physical and cognitive well-being (Kitzman et al. 2017; Langhorne, Bernhardt & Kwakkel 2011). It is aimed at attaining the highest possible level of functional independence, so that patients may be reintegrated into their communities (Kitzman et al. 2017; Langhorne et al. 2011). Previous studies reporting on the function of HIV+ patients post-stroke focussed on mortality rather than their morbidity (Heikinheimo et al. 2012; Hoffmann et al. 2000; Tipping et al. 2007). Furthermore, the outcome measures used to assess function were often global in nature and lacked specificity and sensitivity to describe all aspects of function adequately (Schepers et al. 2007). Previous African studies in Malawi (Heikinheimo et al. 2012) and Cameroon (Mapoure et al. 2019) compared the functional outcome of HIV+ and HIV− stroke inpatients. These studies, as other international studies, used the modified Rankin scale and reported no significant difference in function between the groups. The modified Rankin scale may, however, not be sensitive enough to detect a clinically important change in functional outcomes or reflect the complexities of daily activities as outlined in the World Health Organization’s (WHO) International Classification of health and functioning (Banks & Marotta 2007; WHO 2001). A more recent study by Janse Van Rensberg, Mduzi and Ntsisea (2018), conducted in a rehabilitation centre in South Africa, compared the functional outcome of HIV+ and HIV− stroke patients admitted for inpatient rehabilitation. The researchers used the locally developed Beta assessment tool, which is based on the American version of the Functional Independence Measure. The Beta assessment tool has not yet been validated in a South African stroke cohort, and this study also found no significant difference between HIV+ and HIV− stroke patients. Hence, the aim of this feasibility study was to describe the appropriate methodology for assessing functional outcomes between people with stroke, presenting with varied HIV status in the Western Cape of South Africa.

The objectives of this feasibility study, therefore, were to determine appropriate procedures and potential barriers to participant recruitment, study logistics, data collection and testing procedures, as well as generating preliminary comparative findings in a South African rehabilitation context:

- *Recruitment*: The willingness of clinicians to recruit participants, number of eligible patients, follow-up and drop-out rates, and recommendations for additional strategies.
- *Logistics*: Communication with recruitment assistants and clinicians, scheduling testing dates and times, as well as how to decrease the impact of data collection on each participant’s rehabilitation programme and functioning of the rehabilitation centre.
- *Data collection and testing procedures*: Requirements in terms of space to conduct tests, equipment required for testing, outcome measure utility and documentation.
- Generate preliminary findings on comparisons between the functional challenges of stroke patients with different HIV status.

## Methods

### Setting

The Western Cape Rehabilitation Centre (WCRC) is a specialised rehabilitation centre for persons with physical disabilities. The WCRC is located in Cape Town, and its catchment area comprises the Western Cape and surrounding provinces, including the Northern and Eastern provinces of South Africa. Patients are also referred from surrounding countries such as Lesotho, Zimbabwe and Namibia. The rehabilitation centre treats a range of conditions including stroke. Rehabilitation services include physiotherapy, occupational therapy and speech therapy.

### Site negotiations

In the planning phase, meetings were scheduled with the WCRC management and clinicians. The main outcomes of these meetings were to identify their interest and willingness to participate in the envisaged study and to gain an understanding of the WCRC patient profile, the potential number of stroke patient admissions based on the previous 6 months, as well as the logistics and internal processes needed to successfully recruit and test participants over the study period. In addition, negotiations were made with regard to recruitment and data collection procedures to minimise the impact on clinician administrative tasks.

### Recruitment

Recruitment took place at the WCRC over a 6-month period in 2016. Inclusion criteria were patients aged 18 years and older; who had experienced their first ever stroke; and who were able to respond to verbal cues or commands in English, Afrikaans or isiXhosa. Exclusion criteria were cardiac, renal or liver problems; systemic infection; psychiatric disorders; and previous stroke.

An employee at WCRC was identified as a recruitment assistant and acted as a liaison between the clinicians and the primary investigator (PI). Clinicians would identify potential participants and inform the recruitment assistant. The recruitment assistant would then relay this information to the PI.

### Data collection

Data collection occurred twice a week. The PI would screen patient folders; if all criteria were met, written informed consent was obtained. A data collection sheet was designed and used to retrieve the relevant demographic information and medical history, and a customised scoring sheet was used to collect information for all the functional outcome measures used. Outcome measures included the Barthel Index (BI) along with the use of assistive devices to assess independence in ADL and mobility. The Berg Balance Scale (BBS) was used to assess balance and to determine safety with mobilisation. The BI is considered the gold standard in measuring functional disability in stroke patients and has excellent validity and reliability (Collin et al. 1988; Hsueh et al. 2002; Salter et al. 2013; Wolfe et al. 1991). The 10-item BI was utilised as a self-report measure in this study, with a maximum score of 100 indicating a higher degree of independence in ADL (Salter et al. 2013). Assistive devices issued were also considered a good indicator of function as they provided information on how dependent participants were in terms of mobility.

Berg Balance Scale is considered a strong indicator of independence in ADL (Braun et al. 2016). The BBS was originally designed to measure balance in the elderly but also has good validity and reliability in the stroke population (Berg, Wood-Dauphinee & Williams 1995; Salter et al. 2013). It provides a quantitative assessment of balance and risk of falling with a maximum ideal score of 56, indicating a low risk of falling (Berg et al. 1992).

### Sample size

As this was a feasibility study, a target sample was not an objective. However, for the main study, the sample size was calculated by comparing the distributions of the total score for the BI between the three groups (using Wilcoxon rank-sum test). Assuming a medium effect size of 0.5, power of 80% and a level of significance of 5%, we would need 222 sample participants (74 per group) (Cohen 1969).

### Testing procedures

A separate testing area was made available by the rehabilitation centre. Other than the space and examination table required for testing, the BBS required basic everyday items such as a small step and a ruler. The BI was used as a self-reported measure, and hence, these outcome measures could be considered suitable measures for a low-resource setting. This feasibility study had a pre- and post-design where participants were assessed on admission and reassessed on discharge. To ensure testing did not cause any inconvenience to participants’ rehabilitation schedule, testing times were scheduled once clinicians had set up their daily treatment sessions for all participants. This required the PI to accommodate potential waiting periods between participants’ treatment sessions.

### Planned analysis and evaluation

Recruitment and retention of participants were analysed using methods described by Walters et al. (2017), which determined recruitment rate by dividing the total sample size by months in the recruitment period. Once all data were coded and captured in MS Excel, statistical analysis was conducted. Descriptive statistics were done on demographics and medical history. Continuous data, including BI and BBS, were summarised using median, range and empirical 95% confidence intervals. Statistical analysis was performed using Stata version 14.2 (StataCorp, 2015). Association between categorical variables was assessed using the chi-squared or Fisher’s exact test. Differences in the distribution of continuous variables over different levels of a categorical variable were evaluated using the Kruskal–Wallis (K-W) test, and where differences were detected, the Dunn’s test was used for pairwise comparisons. Statistical significance was assessed at the 5% level.

### Ethical considerations

Approval for the study was obtained from the Health Research Ethics Committee (HREC) at Stellenbosch University (S15/10/232), and permission was also granted by the Western Cape Department of Health. The process of negotiating with the respective site and approval for the study took approximately 10 months prior to the commencement of data collection. To maintain the confidentiality of the HIV status of participants, all stroke patients meeting the inclusion criteria admitted during the data collection period were included in the study. Hence, participants were separated into three subgroups post hoc for analysis: (1) HIV+, (2) HIV− and (3) HIV status unknown.

## Results

### Evaluating feasibility: Recruitment and data collection

**FIGURE 1:**
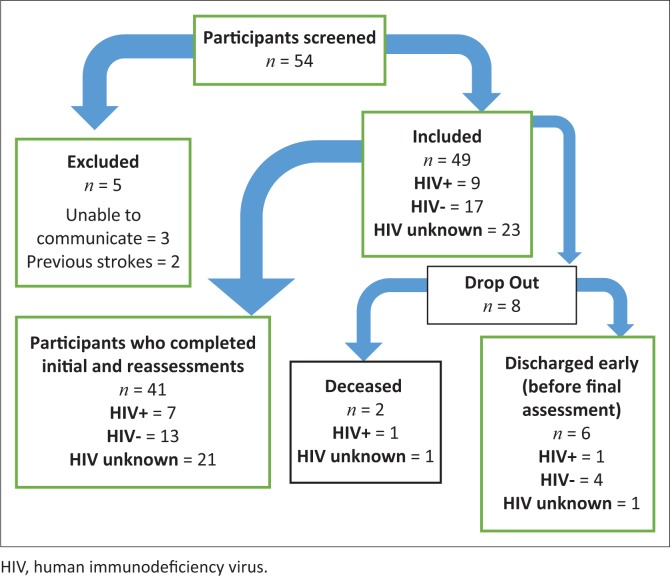
Preliminary findings.

After 6 months of data collection, 54 potential participants were identified. Figure 1 depicts the flow chart of participation. A total of 49 participants were recruited based on inclusion and exclusion criteria. The recruitment rate was eight participants per month, which was lower than expected, particularly for the HIV+ subgroup, in relation to preceding months’ admission statistics and communication with therapists and management of the WCRC. Participants were then separated into their respective subgroups. Table 1 describes participant characteristics in each subgroup to enable further analysis on functional differences.

**TABLE 1:** Demographic and stroke-related characteristics.

Demographic and medical history	HIV+					HIV−				HIV status unknown				Total				Kruskal–Wallis test statistic	Chi-squared (χ^2^)	*p*-value
	*n*	%	Median	IQR	Min–max	*n*	%	Median	IQR	*n*	%	Median	IQR	n	%	Median	IQR			
**Sample size**																				
Admission	9	-	-	-	-	17	-	-	-	23	-	-	-	49	-	-	-	-	-	-
Discharge	7	-	-	-	-	13	-	-	-	21	-	-	-	41	-	-	-	-	-	-
**Age**	-	-	30	27–37	-	-	-	50	46–56	-	-	51	42–62	-	-	48	39–60	10.775	-	0.0046
**Women**	6	66.67	-	-	-	8	47.06	-	-	11	47.83	-	-	25	51.02	-	-	-	0.582	0.607
**Race**																				
Black	6	66.67	-	-	-	4	23.53	-	-	7	30.43	-	-	17	34.69	-	-	-	0.232	0.254
Mixed race	3	33.33	-	-	-	9	52.94	-	-	14	60.80	-	-	26	53.09	-	-	-	-	-
White	0	0.00	-	-	-	3	17.65	-	-	2	8.70	-	-	5	10.20	-	-	-	-	-
Indian	0	0.00	-	-	-	1	5.88	-	-	0	0.00	-	-	1	2.04	-	-	-	-	-
**Type of stroke**																				
Infarction	9	100	-	-	-	14	82.35	-	-	21	91.30	-	-	44	89.80	-	-	-	0.349	0.569
**Risk factors**																				
Hypertension	1	11.11	-	-	-	15	88.74	-	-	20	86.96	-	-	36	73.47	-	-	-	< 0.001	< 0.001
Diabetes	0	0.00	-	-	-	4	23.53	-	-	10	43.48	-	-	14	28.57	-	-	-	0.043	0.042
Cholesterol	1	11.11	-	-	-	2	11.76	-	-	3	13.04	-	-	6	12.24	-	-	-	0.986	1.00
Smoking	2	22.22	-	-	-	7	41.18	-	-	7	30.43	-	-	16	32.65	-	-	-	0.589	< 0.671
Substance abuse	2	22.22	-	-	-	1	5.88	-	-	0	0.00	-	-	3	6.12	-	-	-	0.062	0.038
Opportunistic infections	3	33.33	-	-	-	0	0.00	-	-	0	0.00	-	-	3	6.12	-	-	-	0.001	0.005
CD4 count	-	-	130	-	54–883	-	-	-	-	-	-	-	-	-	-	-	-	-	-	-
**Time: Stroke incident-admission**	-	-	14	21–27	-	-	-	18	16–23	-	-	23	15–28	-	-	21	14–28	1.272	-	0.5293
**Length of stay**	-	-	45	38–51	-	-	-	55	50–59	-	-	53	50–65	-	-	53	46–60	5.402	-	0.0671

*n*, number; IQR, interquartile range; HIV, human immunodeficiency virus.

### Demographics and medical history

The only notable demographic difference between groups was age (*p* = 0.0046), with the median in the HIV+ group at 30 years, in contrast to 50 and 51 years for the HIV− and HIV status unknown groups, respectively (Table 1). With regard to clinical presentation, all those in the HIV+ group sustained an infarction stroke, whereas some incidence of haemorrhagic strokes was noted in other groups (HIV−: *n* = 3 [17.65%] and HIV status unknown: *n* = 2 [8.7%]). Typical risk factors for stroke such as hypertension (*p* < 0.001) and diabetes (*p* = 0.042) were most prevalent in the HIV− and HIV status unknown groups. The HIV+ group, however, had substance abuse (*p* = 0.038) and opportunistic infections (*p* = 0.005) as their more common risk factors (see Table 1). As documented in Table 1, the subgroups were similar with regard to all other characteristics.

### Functional ability and safety

The functional outcome measures utilised have been closely linked to the International Classification of Functioning (ICF) (Stucki, Ewert & Cieza 2003). The ICF is a classification system, which has multiple uses in various sectors and disciplines (WHO 2001). In addition, it describes function at various levels, including activity. The combination of outcome measures utilised in this study gave an adequate description of function with regard to the safety and independence in performing ADL including mobility. Even though there were no significant functional differences between groups on admission or discharge for any of the functional outcomes (ADL, independence and mobility, with *p* = 0.886 [K-W = 0.243]; use of assistive devices, with *p* = 0.300 [K-W = 2.885]; and balance with risk of falling, with *p* = 0.417 [K-W = 1.75]), all groups showed significant improvements. Participants who were HIV+, however, tended to score in the higher percentiles for each functional outcome measure on discharge.

The median BI scores at admission and discharge were similar for all groups (Table 2), but it was smaller for the HIV+ group. On discharge, 17.07% (*n* = 7) of the sample participants were categorised as severely dependent (a score of 21–60), indicative of requiring maximal assistance with self-care and mobility. These participants tended to be older and presented with multiple risk factors. The minimal detectable change (MDC) for the BI is an increase or decrease of 4.02 points (Hsieh et al. 2007). All three groups demonstrated improvement in independence in function after rehabilitation, although the HIV+ group showed less of a median change (Table 2). In contrast, the median difference scores for the HIV− and HIV status unknown groups were more than double the HIV+ group (Table 2).

**TABLE 2:** Independence in activities of daily living, mobility and balance.

Outcome measures	HIV+					HIV−					HIV status unknown					Total					Kruskal–Wallis test statistic	*p*
	*n*	%	Median	IQR	95% CI	*n*	%	Median	IQR	95% CI	*n*	%	Median	IQR	95% CI	*n*	%	Median	IQR	95% CI		
**Sample size**																						
Admission	9	-	-	-	-	17	-	-	-	-	23	-	-	-	-	49	-	-	-	-	-	-
Discharge	7	-	-	-	-	13	-	-	-	-	21	-	-	-	-	41	-	-	-	-	-	-
**Barthel Index**																						
Admission	-	-	50	40–85	40–90	-	-	55	50–75	50–75	-	-	55	40–70	45–80	-	-	55	40–75	53–70	0.416	0.812
Discharge	-	-	95	80–100	80–100	-	-	95	60–100	75–100	-	-	90	75–95	80–95	-	-	90	75–100	80–95	1.184	0.553
Median difference	-	-	10	10–55	10–65	-	-	25	15–40	15–45	-	-	35	20–40	25–45	-	-	35	10–45	25–45	0.243	0.886
**Assistive devices issued**																						
Admission																						
1. No aid required	3	33.33	-	-	-	5	29.41	-	-	-	1	4.35	-	-	-	9	18.37	-	-	-	-	-
2. Walking stick	0	0.00	-	-	-	0	0.00	-	-	-	1	4.35	-	-	-	1	2.04	-	-	-	-	-
3. Crutches	0	0.00	-	-	-	0	0.00	-	-	-	1	4.35	-	-	-	1	2.04	-	-	-	-	-
4. Walking frame	0	0.00	-	-	-	0	0.00	-	-	-	0	0.00	-	-	-	0	0.00	-	-	-	-	-
5. Wheelchair	6	66.67	-	-	-	12	70.59	-	-	-	20	86.96	-	-	-	38	77.55	-	-	-	-	-
Device score at admission	-	-	5	1–5	5–5	-	-	5	1–5	5–5	-	-	5	5–5	5–5	-	-	5	5–5	5–5	2.885	0.236
Discharge																						
1. No aid required	4	57.14	-	-	-	3	25.00	-	-	-	6	30.00	-	-	-	13	33.33	-	-	-	-	-
2. Walking stick	2	28.57	-	-	-	5	41.67	-	-	-	5	25.00	-	-	-	12	30.77	-	-	-	-	-
3. Crutches	0	0.00	-	-	-	0	0.00	-	-	-	1	5.00	-	-	-	1	2.56	-	-	-	-	-
4. Walking frame	0	0.00	-	-	-	0	0.00	-	-	-	0	0.00	-	-	-	0	0.00	-	-	-	-	-
5. Wheelchair	1	14.29	-	-	-	4	33.33	-	-	-	8	40.00	-	-	-	13	33.33	-	-	-	-	-
Device score at discharge	-	-	1	1–2	0–2	-	-	2	2–5	2–5	-	-	2	1–5	2–5	-	-	2	1–5	2–5	2.411	0.300
**Berg Balance Scale**																						
Admission	-	-	27	11–47	11–53	-	-	33	9–49	22–49	-	-	28	7–50	12–44	-	-	29	9–49	24–42	0.362	0.834
Discharge	-	-	54	38–56	7–56	-	-	54	35–54	28–53	-	-	45	34–55	37–55	-	-	47	35–55	35–52	0.724	0.696
Median difference	-	-	8	0–29	0–31	-	-	16	13–22	13–24	-	-	15	4–21	12–28	-	-	15	4–22	13–21	1.75	0.417

*n*, number; IQR, interquartile range; CI, confidence interval; HIV, human immunodeficiency virus.

On admission, the median score for assistive devices issued (median: 5- wheelchair) indicated that majority of participants required wheelchairs as it was not safe for them to mobilise independently; however, no statistical significant difference was found among the groups (*p* = 0.236). On discharge, more than half of the HIV+ group did not require assistive devices as seen in the median discharge score (median: 1 – no aid required) and were able to mobilise unaided, whereas in the HIV− group, 41.67% required a walking stick (median: 2 – walking stick) and 33.33% required a wheelchair. Even though the HIV+ group included participants mostly able to mobilise unaided on discharge (85.71%; *n* = 6), no statistical significant difference was found among the groups with regard to mobility assistive devices at the end of rehabilitation (*p* = 0.300).

Overall, the BBS scores on admission indicated a medium risk of falling (21–40) for the total sample and individual groups alike (Table 2). On discharge, the median BBS score improved with rehabilitation and moved participants into the low risk of falling category (41–56) for both the total sample and the individual groups. The MDC for the BBS is 6.9 points and was surpassed by all groups (Hiengkaew, Jitaree & Chaiyawat 2012). No minimal clinically important difference has been established in the literature as yet (Hiengkaew et al. 2012). Even though the median difference for the HIV− and HIV status unknown groups were double the median difference score of the HIV+ group, no difference was found among groups (*p* = 0.417). Previous studies have developed BBS cut-off scores associated with independence in various ADL (Fujita et al. 2016, 2017). These studies suggest that a BBS score of 40 indicates independence in functional walking ability in strokes; 41 indicates independent transfers; 42 indicates independent toileting; 44 indicates independent dressing; and 54 indicates independence in stair climbing (Fujita et al. 2016, 2017). On admission, median scores suggest that few participants in each group were independent in these ADL (Table 2). On discharge, all groups improved significantly, as indicated in their median change in score. The HIV+ and HIV− groups had a median score of 54, indicating independence in stair climbing. However, the HIV status unknown group had a median score of 45, indicating independence in dressing, but this may indicate that majority of these participants may not have been independent in stair climbing. Nonetheless, this was not significant (*p* = 0.417).

## Discussion

### Barriers to successful study completion

The challenge with ethical approval was ensuring that participant’s HIV status remained unknown to other participants. In addition, if HIV status was at the forefront of the study, potential participants would be reluctant to participate because of the stigma associated with the disease. Hence, all potential participants were included regardless of their HIV status being unknown. The research team was required to budget for travel costs, printing of consent and data collection forms, use of an isi-Xhosa translator when needed and employment of a research assistant. However, to achieve the required number of participants for generalisable results, longer data collection periods and multiple sites would be required. Hence, future researchers should factor in the costing of additional research assistants. Consideration would therefore be needed for the addition of multiple recruitment officers, research assistants and translators at all sites, which would increase the cost involved. In addition, the PI who conducted the testing was not blinded to HIV status, and thus, potential bias was introduced. There was a disproportionate amount of participants in each subgroup. The drop-out rate was mainly affected by participants being discharged earlier than expected. The PI viewed discharge plans as documented in weekly planning by clinicians; these often changed, and the documented plans were sometimes not updated. Because of the dynamic nature of clinical practice and discharge planning, it is recommended that the recruitment assistant be informed of participant discharge planning, or new information should be entered digitally, so that any change in discharge dates are sent via alerts to the research team.

### Potential barriers and preliminary comparative findings

This feasibility study demonstrated objective-function-related description for the larger prospective longitudinal study and also identified challenges future studies would need to accommodate. With regard to recruitment, multiple sites and longer data collection periods are advised to ensure that a suitable sample size is reached. With a recruitment rate of eight participants per month, future studies should prepare for a data collection period of approximately 2 years to achieve an adequate sample size. The selected recruitment sites should be similar in nature. In an inpatient rehabilitation centre, majority of patients were eager to participate as their day-to-day activities consisted of rehabilitation, which is outcome based, making the setting more conducive for this type of research. Future studies should accommodate expected disproportionate amount of HIV+ participants.

The HIV+ group was significantly younger and presented with lower rates of typical risk factors such as diabetes and hypertension associated with ageing, which has been a common trend throughout stroke literature (Heikinheimo et al. 2012; Hu et al. 2005; Jowi, Mativo & Musoke 2007; Mlay & Bakari 2012). Functional recovery time after a neurological injury such as stroke is dependent on neural plasticity, which decreases with age. This is the ability of the brain to learn and relearn function by the adaption of neurons and development of new neural synapses. This in turn expands the amount of motor cortex involved in movement and function (Kleim 2011). As in Janse Van Rensburg et al. (2018), the HIV+ group in this study achieved similar outcomes in a shorter amount of time, with a median length of stay of 45 days, compared to 55 and 53 days in the HIV− and HIV status unknown groups, respectively (*p* = 0.0671). Thus, future studies should look at age-matched subgroups to reduce the heterogeneity between subgroups.

Once the HIV+ participants were medically stable, they made good recovery and scored in the higher percentiles, compared to other groups (see Table 2). However, as in previous studies, this study showed no significant functional differences between groups on admission, or on discharge, for any of the functional outcome measures (see Table 2) (Heikinheimo et al. 2012; Kumwenda et al. 2005; Mapoure et al. 2019; Mlay & Bakari 2012). With previous studies based at acute care facilities and researchers utilising global outcome measures, an appropriate comparison could not be made. The combination of outcome measures used in this study described functional mobility and identified participants’ independence, or assistance required, in performing ADL (Salter et al. 2013). Thus, an adequate description of function with regard to instrumented activities could be produced with these outcome measures. The data collection procedure and combination of measures reported here could easily be utilised in diverse contexts and low-resource settings.

## Conclusion

The combination of outcome measures used in this study provided a good indication of function in terms of ADL, safety and indoor mobility for people with stroke and varying HIV status. These outcome measures provided a good insight into their functional needs and abilities. Future studies should include measures for community re-integration and productive activity to describe the long-term functional outcome of the younger HIV+ stroke population. Even though HIV status seemed not to negatively affect the functional outcome of some stroke patients, results were not generalisable. Keeping in mind the budget and resource implications, future studies should look at larger cohorts with age-matched groups, multiple recruitment sites and longer data collection periods that are required for more generalisable results and to provide a better understanding of the unique functional needs and outcomes of HIV+ stroke patients.
